# Zwitterionic Polysulfone Copolymer/Polysulfone Blended Ultrafiltration Membranes with Excellent Thermostability and Antifouling Properties

**DOI:** 10.3390/membranes11120932

**Published:** 2021-11-26

**Authors:** Dalong Li, Changlu Gao, Xinyue Wang, Gang Wu, Jinghua Yin, Yudong Huang, Xiuhua Sun

**Affiliations:** 1 School of Marine Science and Technology, Harbin Institute of Technology at Weihai, Weihai 264209, China; changlugao@163.com (C.G.); hitwhwxy@163.com (X.W.); 2 Shandong Weigao Group Co., Ltd., Weihai 264210, China; wugwego@163.com (G.W.); yinjhwego@163.com (J.Y.); 3 National Engineering Laboratory of Medical Implantable Devices & Key Laboratory for Medical Implantable Devices of Shandong Province, WEGO Holding Company Limited, Weihai 264210, China; 4 School of Chemical Engineering and Technology, Harbin Institute of Technology, Harbin 150001, China; huangydhit@163.com

**Keywords:** sulfobetaine polysulfone, zwitterions, antifouling, thermostability

## Abstract

Membrane fouling has been one of the most important challenges in membrane separation operations. In this study, we report a facile strategy to prepare antifouling polysulfone (PSf) UF membranes by blending amphiphilic zwitterion polysulfone-co-sulfobetaine polysulfone (PSf-co-SBPSf) copolymer. The copolymer chemical structure was characterized by ^1^HNMR spectroscopy. The PSf/PSf-co-SBPSf blend membranes with various zwitterionic SBPSf segment contents exhibited better surface hydrophilicity and excellent antifouling ability compared to PSf and PSf/PEG membranes. The significant increase of both porosity and water permeance indicates that the PSf-co-SBPSf has a pore-forming effect. The pure water flux and flux recovery ratio of the PSf/PSf-co-SBPSf blend membranes were both remarked to improve 286.43 L/m^2^h and 92.26%, while bovine serum albumin (BSA) rejection remained at a high level (97.66%). More importantly, the water flux and BSA rejection see minimal variance after heat treatment, indicating excellent thermostability. Overall, the PSf/PSf-co-SBPSf blend membranes achieved a comprehensive performance of sustainable hydrophilic, high permeation flux, and remarkable antifouling ability, thus becoming a promising candidate in high-temperature separation application.

## 1. Introduction

Membrane filtration is a green separation method with numerous applications due to its energy efficient, environmentally friendly and modular separation in comparison with other separation process [1,2,3]. Among the various separation membranes, ultrafiltration (UF) membranes show unique advantages in terms of the rapid removal of colloid particles, macromolecular protein, viruses, and bacteria [4,5,6]. The polysulfone (PSf) matrix membranes are commonly used for UF membranes, as well as support membranes for microfiltration, nanofiltration and reverse osmosis, due to their strong mechanical properties, thermal and chemical stability [7,8,9,10]. However, the PSf matrix UF membranes’ intrinsic hydrophobic nature makes it easy to adhere to protein and bacteria, which leads to membrane fouling [11,12,13]. Fouling results in a decline in flux, leading to reduced productivity and increased energy costs [14,15,16,17,18]. Therefore, developing fouling-resistant membranes is of great important in filtration.

To achieve the above pursuit, various strategies, such as surface modification, feed pretreatment and innovation, have been adopted to alleviate membrane fouling [14,19,20,21,22]. Polymer blending modification as an improvement strategy endows the PSf membranes with better antifouling ability, which is considered as one of the most promising technologies to develop antifouling membranes [23,24]. Among the commercially available hydrophilic polymers, poly(vinylpyrrolidone) (PVP) and poly (ethylene glycol) (PEG) have been widely used as blending additives in fabricating antifouling membranes due to their non-toxicity, low cost, and excellent hydrophilicity [25,26,27]. However, PEG and PVP blending membranes tend to lose PVP or PEG gradually in long-term filtrations due to their linear structures [26,28]. This phenomenon leads to a reduction in hydrophilicity and antifouling performance, and even contamination of the filtered solution [25]. To solve this problem, amphiphilic copolymers consisting of hydrophobic backbones and hydrophilic segments have been developed to replace linear hydrophilic copolymers [28,29]. These amphiphilic copolymers exhibit excellent antifouling properties and pore-forming effects, resulting from the distribution of hydrophilic segments onto the membrane surface and internal wall. Meanwhile, the hydrophobic backbones could provide compatibility of copolymers with polymer matrix, which anchors with the polymer matrix to prevent the leaching out of the amphiphilic segment, leading to long-term stability of membrane [28,29,30]. At present, most work on copolymer blending focused on searching for new copolymers with better solubility and antifouling properties.

Zwitterions have emerged as a new generation of antifouling materials because of their strong intrinsic hydrophilicity, which forms a hydration layer through electrostatic interaction between water molecules and zwitterions [31,32,33]. The hydration layers provide a repulsive force to effectively decreases biofoulant adsorption or attachment on the substrate surface [31,33]. Polysulfobetines, in which both the sulfonate anion and ammonium cation are covalently attached to the same repeat unit, have been most commonly used to prepare zwitterionic polyelectrolytes that improve the antifouling properties of separation membranes [32,34,35]. As a result, the direct blending of polysulfobetain-based polymers for antifouling membrane has been widely reported. However, the amount of zwitterionic polymers exhibits weak miscibility with organic casting solutions due to their special structure and exclusion of hydrophilic polymers and hydrophobic polymers. [36,37]. Therefore, the blend amount of a zwitterionic copolymer would be limited, which would affect the antifouling performance of UF membranes. The grafted zwitterionic polymers, such as polysulfone-graft-poly (sulfobetaine methacrylate) (PSf-g-PSBMA), had better chemical stability, good hydrophilicity and the activity of reducing adhesion of microorganisms on the membrane surfaces, but the preparation methods were complicated and hard to industrialize [38]. All in all, considering its unique antifouling performance and low cost for large-scale membrane preparation, it is necessary but still a challenge to develop zwitterionic antifouling membranes via more simple and effective methods.

**Scheme 1:**
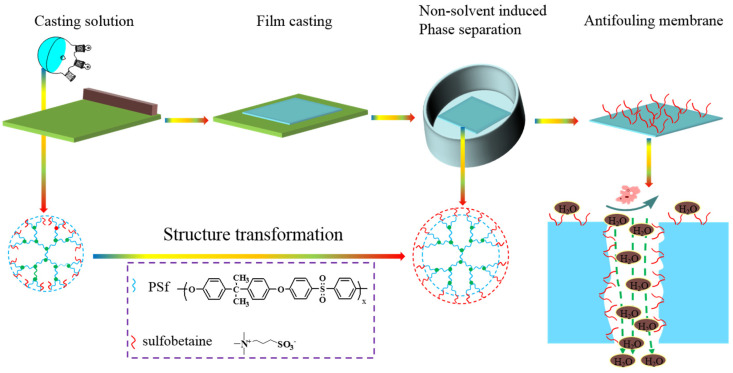
Schematic diagram of the fabrication process of zwitterionic surface antifouling membrane.

In this work, a new zwitterionic amphiphilic copolymer polysulfone-co-sulfobetaine polysulfone (PSf-co-SBPSf) was synthesized via a facile synthesis route, and a series of charge-modified polysulfone-based antifouling membranes with blending PSf-co-SBPSf were prepared by the non-solvent-induced phase separation (NIPS) method (Scheme 1). The zwitterionic amphiphilic copolymer, polysulfone-co-sulfobetaine polysulfone (PSf-co-SBPSf), had better stability in blend membranes compared with the linear copolymer. Moreover, the excellent compatibility between PSf-co-SBPSf and PSf made the blend system stable during preparation and use. The prepared blending membranes were characterized by proton nuclear magnetic resonance (^1^HNMR), scanning electron microscopy (SEM), X-ray photoelectron spectroscopy (XPS), and water contact angle. The effects of zwitterionic SBPSf segments on membrane morphology, separation performance and antifouling properties were also systematically investigated. To our best knowledge, the amphiphilic copolymer, with polysulfone-based bulk zwitterion and blended with polysulfone, will be rare and very promising. The amphiphilic polymer-blended PSf membranes are expected to have low fouling propensities and thermostability.

## 2. Materials and Methods

### 2.1. Materials and Reagents

Bisphenol A (BPA, 99%), dimethylamine (40%), polyethylene glycol (PEG, Mn~400 g/mol), potassium carbonate (K_2_CO_3_, ≥99%) were obtained from Aladdin. The 4,4-difluorodiphenylsulfone (DFDPS), 1,3-propane sultone, bovine serum albumin (BSA, ≥98%), deuterated dimethyl sulfoxide (DMSO-d6) and deuterated chloroform (CDCl_3_) were purchased from Sigma-Aldrich. N-Methyl-2-pyrrolidone (NMP), toluene, formaldehyde, Na_2_HPO_4_·12H_2_O, KH_2_PO_4_·2H_2_O were all purchased from Aladdin. Polysulfone (PSf, Mn~80,000 g/mol) was provide by Solvay, China.

### 2.2. Synthesis of PSf-Co-SBPSf Copolymers

#### 2.2.1. Synthesis of Bisphenol A with Pendant Tertiary Amine Groups

**Scheme 2:**
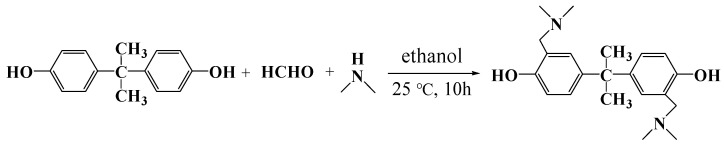
Reaction route of 2,2-dimethylaminemethylene-4,4-biphenol A.

The bisphenol A monomer with two pendant tertiary amine groups was synthesized by the Mannich reaction [39]. Firstly, bisphenol A (22.83 g, 0.1 mol) was dissolved in 300 mL ethanol in a 500 mL round-bottomed flask, and then aqueous solutions of dimethylamine (11.27 g, 0.25 mol, 40%) and formaldehyde (9.0 g, 0.3 mol, 37%) were added. The mixed solution was stirred at room temperature for 10 h. Then, the solution was evaporated under low pressure to obtain crude products. The silica gel chromatography was used to purify the crude with ethanol as the solvent. Finally, the purified product was dried at 60 °C under vacuum for 24 h, and named as 2,2-dimethylaminemethylene-4,4-bisphenol (DABA). The reaction route is shown in Scheme 2.

#### 2.2.2. Synthesis of PSf-Co-Tertiary Amine-Modified PSf (PSf-Co-TAPSf) Copolymer

The PSf-co-TAPSf copolymer was synthesized via traditional step-growth polymerization [40]. The reaction was carried out in a three-neck flask equipped with a Dean Stark trap, a condenser, nitrogen inlet/outlet, and a mechanical stirrer. Firstly, the reactor flask was purged with nitrogen for 15 min. Then, bisphenol A (4.79 g, 21 mmol), DABA (3.08 g, 9 mmol), DFDPS (7.629 g, 30 mmol), and K_2_CO_3_ (4.98 g, 36 mmol) were added into the three-necked flask. Subsequently, NMP (80 mL) and toluene (50 mL) were introduced as a solvent and azeotrope reagent. The reaction was heated under reflux at 120 °C for 4 h while the toluene-water was removed from the reaction mixture. Next, the reaction was gradually heated to 150 °C and further reacted for another 8 h. The reaction mixture was cooled to room temperature and poured into deionized water. The polymer was washed with deionized water and ethanol several times. Finally, the polymer was dried at 100 °C under vacuum for 24 h.

#### 2.2.3. Synthesis of PSf-Co-SBPSf Copolymers

**Scheme 3:**
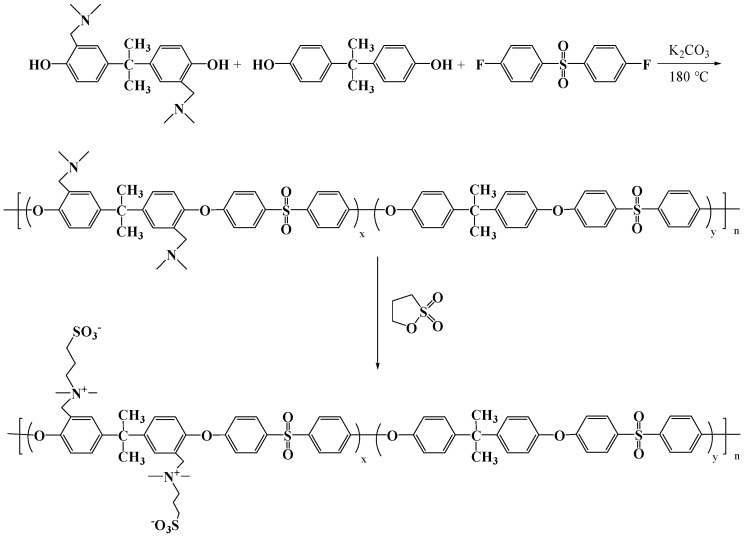
Synthesis of PSf-co-SBPSf copolymers.

To a solution of PSf-co-TAPSf (5.1 g, 0.5 mmol) in CHCl_3_ (100 mL), 1,3-propane sultone (366.5 mg, 3 mmol) was added. The solution was stirred at 60 °C for 48 h. The copolymers were obtained by rotating evaporation. In addition, to demonstrate the solubility of the zwitterionic copolymer, a different proportion of TAPSF copolymer was synthesized and functionalized by controlling the DABA:BPA ratio in the same fashion as described in Section 2.2. In this case, the different proportion of TAPSf copolymer was named as PSF-co-SBPSF (I) with DABA:BPA:DFDPS 1:9:10, PSF-co-SBPSF (III) with DABA:BPA:DFDPS 3:7:10, and PSF-co-SBPSF (VI) with DABA:BPA:DFDPS 6:4:10. The best balance was achieved with 30% TAPSf copolymer. The synthetic route is shown in Scheme 3. (If no special illustration, the PSf-co-SBPSf copolymer stands for the PSf-co-SBPSf (III) in this study).

### 2.3. Characterization of PSf-Co-SBPSf Copolymers

The structures of the pendant tertiary amine functionalized bisphenol A, PSf-co-TAPSf and PSf-co-SBPSf copolymers were characterized by ^1^H NMR spectroscopy. The spectra were recorded on a Bruker 400 MHz NMR (ADVANCE III, Karlsruhe, Germany) spectrometer using DMSO-d6 and CDCl_3_ as the solvent.

### 2.4. Membrane Preparation

PSf/PSf-co-SBPSf UF membranes were prepared by non-solvent-induced phase separation (NIPS). In a typical process, a certain amount of PSf-co-SBPSf (2%, 3%, 4%, 5%) was well-dispersed into NMP with ultrasonic treatment for 30 min, respectively (labeled as M2, M3, M4, M5). Then, the uniform suspension was mixed with PSf (15%) under continuous stirring at 60 °C for 24 h. Meanwhile, pristine PSf (M0) and blended membrane with PEG (M1) were prepared and compared regarding properties. All casting solution was kept in a vacuum oven to remove air bubbles at 60 °C for 24 h. After degasification, the homogeneous dope solution was cast on a glass plate with 150 µm thickness, and subsequently immersed into a coagulation bath for the wet-phase inversion step. Finally, the resulting UF membranes were kept in deionized water for at least 24 h to remove the residual solvent. The various compositions of all membrane dope solutions are summarized in Table 1.

### 2.5. Characterization of the Membranes

The cross-section morphology of the prepared blending membrane was observed by scanning electron microscopy (SEM, Nova Nanosem 450, Brucke, Germany). Membrane samples were freeze-fractured using liquid nitrogen for cross-sectional examination, and sputter coated with gold before imaging.

The surface chemical composition of the blended membrane was analyzed by X-ray photoelectron spectroscopy (XPS, PHI 5400 ESCA system, New York, NY, USA) using Al Kα (*E* = 1486.6 eV) as a radiation source (the take-off angle of photoelectron was set to 90°). The N 1s core-level peaks of hybrid membranes were fitted by using a Lorentzian–Gaussian mixed function.

The surface hydrophilicity of the dried membranes was tested by water contact angle measurement (JGW-360 A, Jinan, China). The test was performed by depositing 2 μL water drops on the top surface of the dry membrane. Five random spots were measured for each membrane at room temperature and the average value was taken.

The porosity and pore size of each membrane was determined through the dry–wet method [41,42]. Membrane samples were immersed in deionized water for 24 h, and then weighted after removal of excess water from the surface by wiping with filter paper. The wet membranes were dried after weighing in a vacuum at 50 °C for 12 h. The dried membranes were weighted. The porosity of the membranes was obtained by Equation (1):

(1) $$\varepsilon =\frac{\left(m_{1}-m_{2}\right)/\rho_{w}}{\left(m_{1}-m_{2}\right)/\rho_{w}+\left(m_{1}/\rho_{p}\right)}$$

where *ε* is the porosity, *m*_1_ is the weight of the wet membrane (g), *m*_2_ is the weight of the dry membrane (g), *ρ_w_* is the water density (1.0 g/cm^3^, 25 °C), and *ρ_p_* is the polymer density (1.25 g/cm^3^, 25 °C).

Furthermore, the mean pore size (*r_m_*) was calculated by the Guerout–Elford–Ferry Equation [41,43]:

(2) $$r_{m}=\sqrt{\frac{\left(2.9-1.75\varepsilon \right)8\mu lJ}{\varepsilon \Delta P}}$$

where *ε* is the porosity (%), *μ* is the water viscosity (8.9 × 10^−4^ Pa s), *l* is the membrane thickness (m), *J* is the flux (m^3^ h^−1^ m^−2^), and Δ*P* is the operation pressure (Pa).

Filtration experiments were performed on 25 mm diameter membranes using a 150 mL dead-end filtration cell with an effective filtration area of 12.56 cm^2^. The membrane samples were pre-compacted with deionized water for 30 min at a pressure of 0.1 MPa to reach a stable flux. Upon stabilization, pure water flux was collected at 5 min intervals for an hour at a pressure of 0.1 MPa and the average water flux was recorded as *J_w,1_*, which was calculated using Equation (3):

(3) $$J_{w,1}=\frac{V}{A\times \Delta t}$$

where *J_w,1_* is the pure water flux (L m^−2^ h^−1^), *V* is the volume of water permeated (L), *A* is the effective membrane area (m^2^) and ∆*t* is the permeation time (h).

To characterize the rejection performance of UF membranes, we used bovine serum albumin (BSA) as the foulant solution during filtration tests. A 1.0 g/L BSA solution (PBS, pH 7.4) was filtered through the membrane immediately after the pure permeance test. The BSA rejection was collected at 5 min intervals for an hour at 0.1 MPa and the BSA flux was recorded as *Jp*. The BSA rejection ratio (*R*) was determined using the following Equation (4):

(4) $$R=(1-\frac{C_{1}}{C_{0}})\times 100\%$$

where *C*_0_ and *C*_1_ are the BSA concentrations in feed and permeate solutions (mg/mL), respectively, as measured by a UV-vis spectrometer (UV1800PC, Beijing, China) at a maximum absorption at 280 nm.

After BSA solution filtration, the membrane was rinsed with deionized water for 30 min. Then, pure water flux was measured again and recorded as *J_w,2_* to determine the flux recovery ratio (*FRR*). Each membrane was measured during 3 cycles to evaluate the antifouling performance. The FRR was calculated using the following Equation (5):

(5) $$FRR=\frac{J_{w,2}}{J_{w,1}}\times 100\%$$

To further analyze the membrane fouling resistance process in detail, the total fouling ratio (*R_t_*), reversible fouling ration (*R_r_*), irreversible fouling ration (*R_ir_*) were calculated using Equations (6)–(8):

(6) $$R_{t}=(1-\frac{J_{p}}{J_{w,1}})\times 100\%$$

(7) $$R_{r}=(\frac{J_{w,2}-J_{p}}{J_{w,1}})\times 100\%$$

(8) $$R_{ir}=(\frac{J_{w,1}-J_{w,2}}{J_{w,1}})\times 100\%$$

All the ultrafiltration and antifouling results in this study are the average values obtained by three measurements from three membrane samples.

### 2.6. Membrane Thermostability Tests

To test the thermostability of the membrane under high temperature, the M0, M1 and M5 membranes were immersed into hot water at 80 °C for up to 12 h. Subsequently, these membranes were rinsed with deionized water twice. Then, the surface hydrophilicity and pure water flux of ultrafiltration membranes were measured again.

## 3. Results and Discussion

### 3.1. Synthesis and Characterization of PSf-Co-SBPSf Copolymers

The bisphenol A monomer with two pendent tertiary amine groups was synthesized via the Mannich reaction and purified by silica gel chromatography (Scheme 1). Copolymers containing a relatively hydrophobic PSf backbone and hydrophilic sulfobetaine side chains were synthesized by step polymerization and post-polymerization modifications (Scheme 2). The backbone structure was chosen as PSf due to its compatibility and strong mechanical strength [9,44]. Sulfobetaine was attached to the PSf backbone as a functional group due to its hydrophilicity and demonstrated anti-fouling performance [33,45]. Moreover, the concentration of sulfobetaine can be tailored by adjusting the ratio of DABA/BPA. Finally, the blended PSf-co-SBPSf membranes were obtained with a PSf matrix with tunable charge content. Two factors would affect the membrane properties: The copolymer chain length and the fraction of charge [46,47]. Although the higher concentration of zwitterion copolymers has better antifouling performance, it has a poor solubility in organic solvent (i.e., free-standing membrane). In our study, the best balance was achieved with 30% TAPSf copolymer.

**Figure 1:**
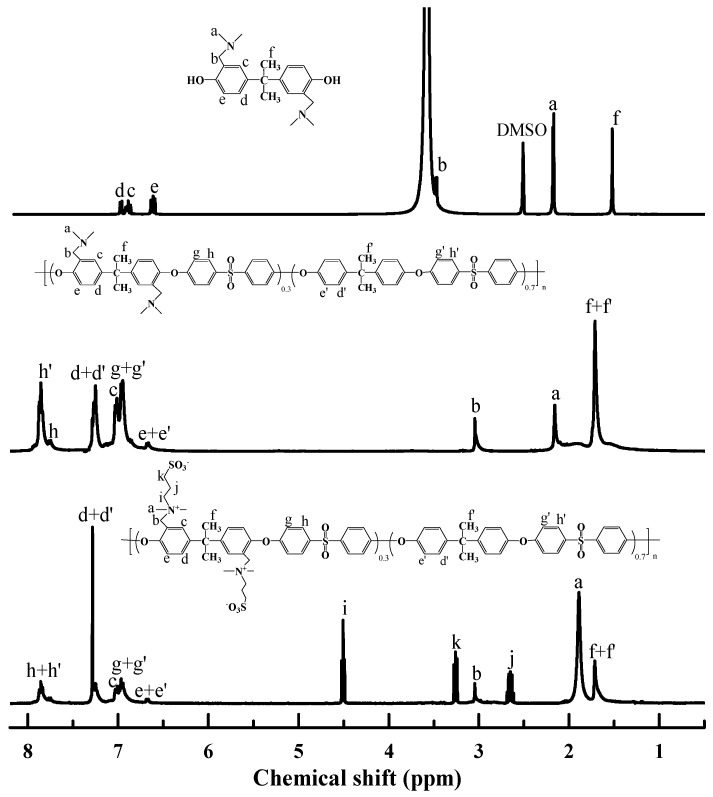
^1^H NMR spectrum of the DABA in DMSO-d6 (**top**), PSf-co-TAPSf (**middle**), and PSf-co-SBPSf (**bottom**) in CDCl_3_, respectively.

To verify the chemical structure of synthesized DABA and copolymers, ^1^H NMR spectroscopy was conducted and shown in Figure 1. The ^1^H NMR spectrum showed peaks consistent with the corresponding structure of bisphenol A. The characteristic peaks of pendent tertiary groups were observed at about 2.25 ppm. Compared to PSf-co-TAPSf copolymer, the PSf-co-SBPSf copolymer appeared 4.52, 3.26, 2.65 ppm peaks, which was attributed to the sulfobetaine groups [48]. From the analysis of ^1^HNMR spectra, the ratio of DABA incorporated into the PSf-co-SBPSf copolymer matched what was fed to the reaction. Therefore, amphiphilic PSf-co-SBPSf with SBPSf content of 30% was synthesized successfully.

### 3.2. Membrane Characterization

#### 3.2.1. Membrane Morphology

**Figure 2:**
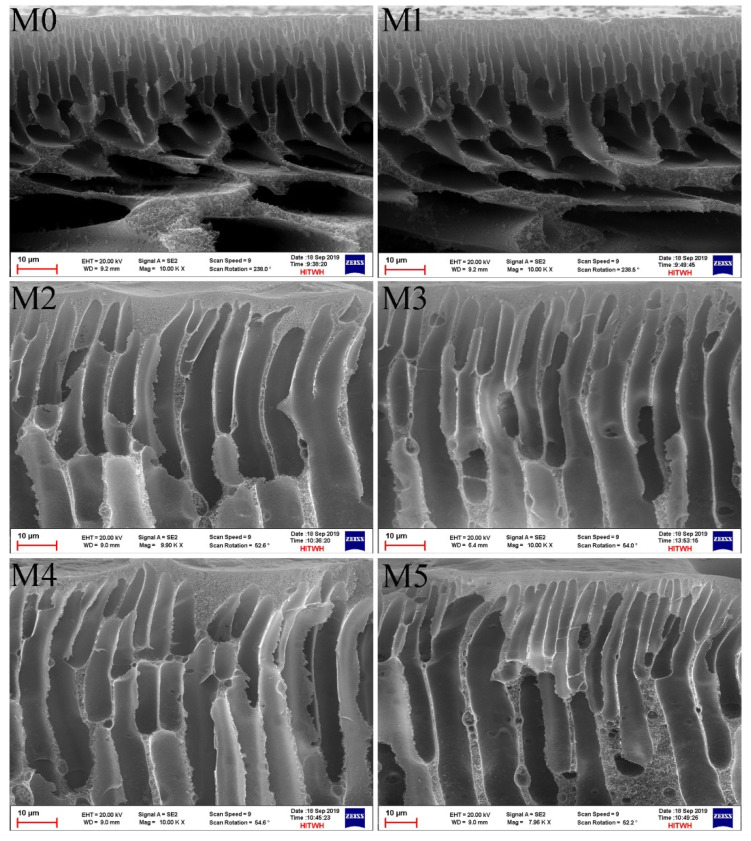
The SEM images of PSf (M0), PSf/PEG (M1), and PSf/PSf-co-SBPSf blend membranes (M2–M5).

To study the morphology of the membranes as a function of zwitterion content in the blend polymer, the cross-sectional structures of the pristine PSf and blend membranes with varying SBPSf contents were characterized by SEM. The porosity and pore size were calculated using Equations (1) and (2). As shown in Figure 2, all of the membranes showed typical asymmetrical structures, consisting of a dense selective layer on the top surface and a porous sublayer beneath as finger-like cavities at the bottom layer. The dense skin layer contributes to high foulant rejection and finger-like cavities should aid water transmission. Compared with pristine PSf and PSf/PEG membranes, obvious differences could be observed that the finger-like porous structures in the cross-section became more visible and the macrovoid gradually developed with the increasing zwitterion content in blend membranes. This was possibly attributed to the instantaneous exchange between solvent and coagulation by the decrease in the viscosity of the blend membrane solution [49]. The result was consistent with the porosity of the membranes. The mean pore size and bulk porosity of all membranes were listed in Table 2. Adding zwitterionic copolymer increased the bulk porosity and mean pore size of the membranes. The pristine PSf membrane exhibited the smallest bulk porosity and mean pore size, which were 46.3% ± 0.6% and 3.9 nm, respectively. However, the bulk porosity of the blend membrane reached 85% for the M5 membrane. The PSf/PSf-co-SBPSf blend membranes have more microvoid and higher porosity than the PSf/PEG membrane, indicating excellent pollutant rejection capacity. The membrane porosity increased with SBPSf content, which indicates that amphiphilic zwitterion copolymers not only improve hydrophilicity, but also played a role as pore-forming agents.

#### 3.2.2. Membrane Surface Element Analysis and Hydrophilicity

**Figure 3:**
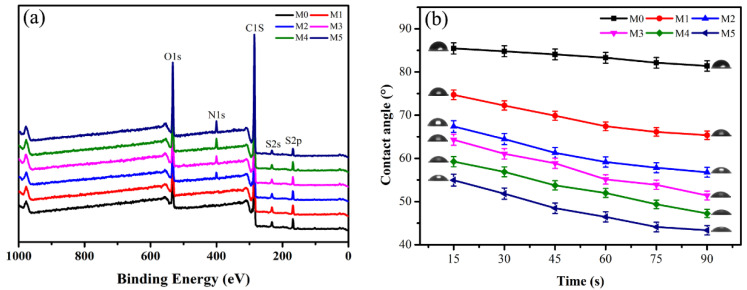
The XPS spectra (**a**) and time-dependent surface water contact angle (**b**) of UF membranes.

The surface chemical composition of UF membranes plays an important role in enhancing hydrophilicity and antifouling. In order to further confirm the surface enrichment of zwitterionic hydrophilic segment, the surface chemical compositions of the pristine PSf membrane and the blend membranes were characterized by XPS. As shown in Figure 3a, compared with the pristine PSf membrane (M0) and PSf/PEG membrane (M1), all PSf/PSf-co-SBPSf blend membranes exhibit an additional N 1s peak with binding energy of 399.1 eV. Moreover, the intensity increases for N 1s and O 1s peaks with the PSf-co-SBPSf ratio in casting solution. This implies that the sulfobetaine segment was successfully anchored onto the PSf backbone, and the growing enrichment on the membrane surface was realized during phase inversion.

Surface hydrophilicity was one of the most important factors affecting the membrane antifouling. Water contact angle (WCA) measurement was used to characterize the surface wettability and hydrophilicity. Typically, a smaller contact angle value corresponds to a better hydrophilic surface [50]. Figure 3b describes water contact angles of the pristine PSf membrane and blend membranes. The pristine PSf membrane is up to 85.46°, indicating the hydrophobic property of the PSf membrane. As expected, the WCA of blend membranes decreased gradually with the increase of PEG and PSf-co-SBPSf segment contents, suggesting improved hydrophilicity. The blend membrane of 5% PSf-co-SBPSf (M5) showed the lowest WCA (54.95°), when compared to the pristine PSf membrane and PSf/PEG membrane. This phenomenon is caused by the segregation toward the membrane surface of the hydrophilic segments of PSf-co-SBPSf, and the strong interaction between the zwitterionic groups and water. The phenomenon indicates that zwitterion copolymer has better hydrophilic properties than PEG. The WCA of all membranes decreases with time, and the blended membranes decreasing more indicates better hydrophilic properties. The result further proves zwitterion hydrophilic segments migrate to the membrane surface, which was in agreement with the surface composition analysis by XPS.

### 3.3. Permeation and Separation Properties of the Membranes

**Figure 4:**
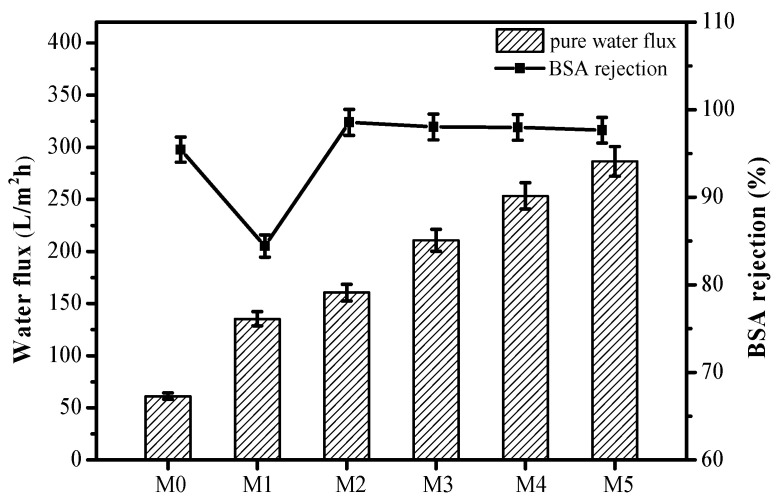
Water fluxes and BSA rejection of the ultrafiltration membrane.

To compare separation performance of the pristine PSf membrane and blend membranes, filtration experiments of pure water and BSA solution were performed. As shown in Figure 4, these results suggest that the blend membranes led to an increase in water permeation flux compared to the pristine PSf membrane under a feed pressure of 0.1 MPa. The pristine PSf membrane had a small water flux of 60.28 L/m^2^h, and the BSA rejection was 95.43%. However, the pure water flux and BSA rejection of blend membranes were greatly enhanced with the increase of PSf-co-SBPSf copolymer concentration. For the M5 membrane, the water fluxes up to 286.43 L/m^2^h, which was almost five times that of the pristine PSf membrane, and the BSA rejection exhibited a slight increase (97.66%). This could be ascribed to the increase in the membrane porosity and the decrease in the thickness of the dense skin layer. Furthermore, the trade-off relationship between permeability and selectivity was broken by blending PSf-co-SBPSf copolymers. This is due to the fact that the zwitterion amphiphilic copolymer tends to produce more narrow pore channels, as summarized in Table 2. Although the PSf/PEG blend membrane also exhibited increased water flux, the BSA rejection rate was significantly reduced (84.4%). Therefore, the zwitterion copolymer is more suitable as a pore-forming agent for industrial application.

### 3.4. Antifouling Performance of the Membranes

**Figure 5:**
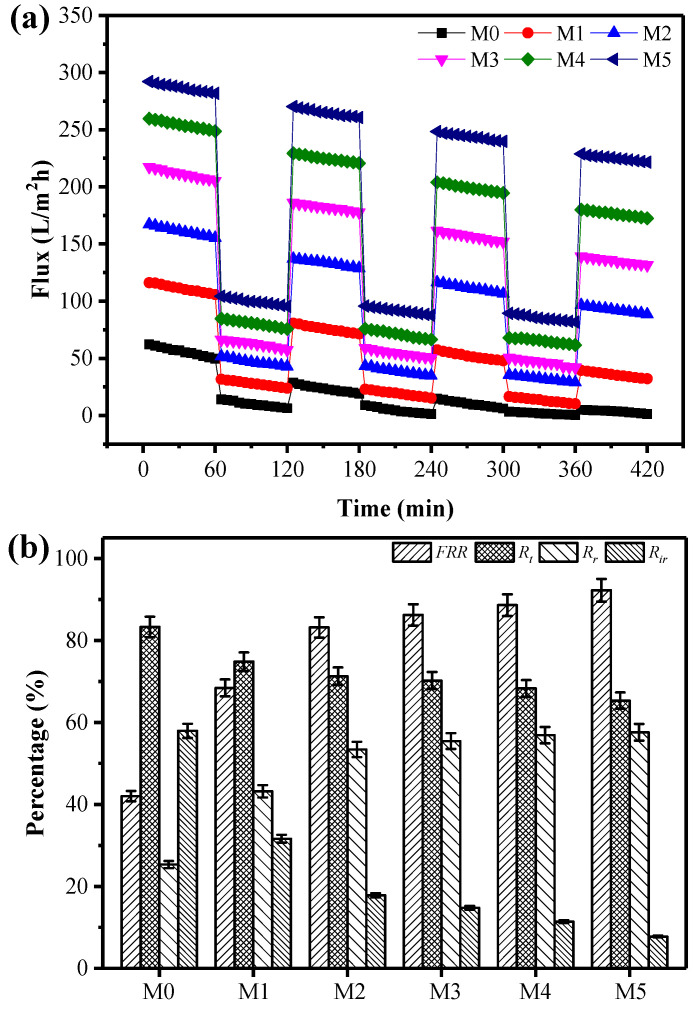
Fouling resistance of membranes: (**a**) time-dependent flux of membranes with BSA as pollutant, (**b**) summary of FRR, R_t_, R_r_ and R_ir_ of membranes with BSA as a pollutant.

Anti-fouling performance is an important property of UF membranes in the separation and purification, which indicates the reusability. The fouling measurement was performed by the three-cycle filtration experiments. The resulting time-dependent flux is shown in Figure 5a. Firstly, pure water and BSA solution were filtrated across the membranes for 1 h, respectively. According to the time-dependent flux dates, when the pure water solution was changed to BSA pollutant solution, flux values of all the membrane had a sharp drop. The average pure water flux of the M5 membrane is 286.43 L/m^2^h, while the average BSA flux is only 103.26 L/m^2^h. Then, the membranes were cleaned for 30 min with water and filtrated with pure water once again. The pure water flux of all membranes after cleaning could not be restored completely to its initial value after three cycles of filtration tests. This is due to the fouling caused by the deposition and adsorption of the BSA onto the membrane surfaces and within the membrane pores, which were difficult to clean completely by washing. However, the pure water flux recovery for the blend membranes was found to be greater than the pristine PSf membrane, showing the higher antifouling performance.

To further investigate the antifouling properties of the membranes with BSA solution in detail, FRR, R_t_, R_r_ and R_ir_ values were calculated, respectively, and presented in Figure 5b. The FRR for the M5 membrane was 92.26%, which is much higher than that of pristine PSf membrane of 42.03% and PSf/PEG blend membrane of 68.43%, indicating their excellent antifouling ability and reusability. There was also an obvious decline in R_t_ values between PSf/PSf-co-SBPSf and PSf/PEG blend membranes, and the values reduced more as the zwitterion concentration increase. That could be attributed to greater coverage of sulfobetaine groups on the membrane surface. Additionally, the R_r_ value of all blend membranes is higher than pristine PSf membrane, and as high as 57.6% for M5 membrane, which weakens interactions between foulant and membrane surface and could be removed by simple washing as a result of antifouling and reusability [14,51]. In contrast, R_ir_ of the blend membrane M5 is 7.74%, which is also dramatically lower than that of the pristine PSf membrane, at 57.96%. A relatively lower R_ir_, the reusability of blend membranes, was further proven [52]. Based on these results, it can be concluded that the introduction of PSf-co-SBPSf in blend membranes effectively prevents protein adsorption, as manifested by the high FRR and low R_ir_ values. The structure of the copolymer provides long-term stability of the membranes and also confers excellent hydrophilicity and fouling resistance to the membranes. The excellent antifouling ability of the blend membranes was further illustrated with antibacterial tests. SEM images show that more *E. coli* bacteria adhered on the surface of the pristine membrane. However, only a few bacteria can be observed on the surface of the blend membranes (Figure S1).

### 3.5. Thermostability Performance of the Membranes

**Figure 6:**
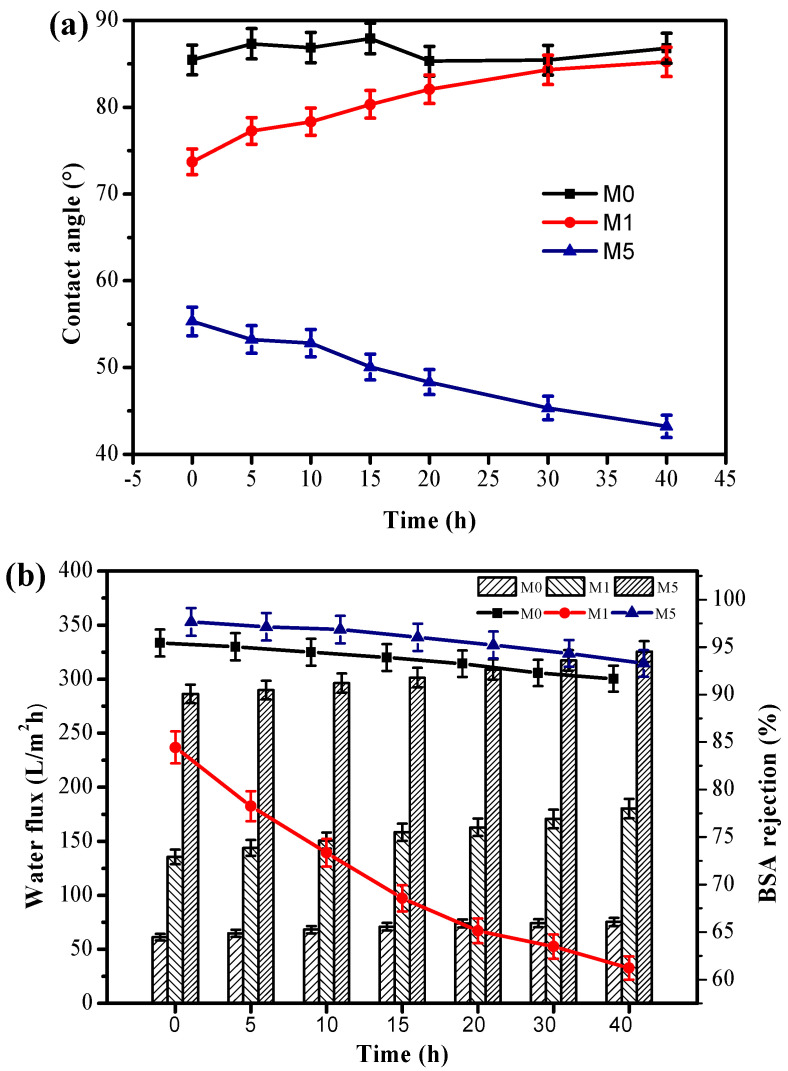
The effect of heat treatment (80 °C) on water contact angle (**a**), water flux (bar graphs) and BSA rejection (line graph) (**b**) in different incubation periods.

For antifouling UF membranes, the sustainable hydrophilicity, stable water flux and pollutant rejection were key important factors in the practical application. To explore the stability of copolymer additive in blend membrane matrix, UF membranes were heated in hot water (80 °C) for a certain time. The values of water contact angle, water flux and BSA rejection of heated membranes were record with time. It has been reported that when polymer matrix was blended with PEG, there is inevitable leakage from the membrane matrix during separations, resulting in reduced hydrophilicity [53]. As shown in Figure 6, this study further proves the conclusion. For the PSf/PEG blend membrane, WCA and water flux have a sharp increase with time after heat treatment, and the BSA rejection decreased significantly. The increase in the WCA and flux is due to the loss of PEG, whereas the decrease of BSA rejection is consistent with the trade-off relationship between the flux and rejection. The results indicate the thermodynamic instability of the PSf/PEG blend membrane. However, the PSf/PSf-co-SBPSf blend membranes have a significant decrease in WCA, and slight change in water flux and BSA rejection. The decrease of WCA was due to more zwitterion segments migrating onto the membrane and pore channel surfaces after heat treatment. The slight change could be attributed to increase the mobility of water molecules at high temperature. Clearly, the zwitterionic PSf have the potential to perform as antifouling separation membrane materials for high-temperature application.

## 4. Conclusions

In this work, we reported a facile approach to prepare novel polysulfone-based ultrafiltration membranes via NIPS with antifouling and thermostability properties by blending PSf-co-SBPSf copolymer. The zwitterionic PSf-co-SBPSf copolymer was synthesized via the typical Mannich reaction and direct step polymerization method. Compared to the PSf and PSf/PEG membranes, the hydrophilicity, separation performance and antifouling ability of PSf/PSf-co-SBPSf blend membranes were improved remarkably due to the decrease of the skin layer and the increase of the porosity. The result illustrated that the PSf-co-SBPSf could be as a novel porogen, and hydrophilic segment emigrated to the membrane surface during phase inversion. More importantly, the performance of PSf/PSf-co-SBPSf blend membranes has hardly changed after heat treatment. As a control, the PSf/PEG blend membrane exhibited significantly reduced BSA rejection and antifouling ability due to PEG loss at high temperatures. This indicates that the PSf/PSf-co-SBPSf blend membranes have more potential in practical application. Furthermore, the adjusting of the charge content and chain length in the copolymer will provide a great prospect for the antifouling UF membranes. The study provides important research value for the high-temperature application of separation.

## Figures and Tables

**Table 1:** Composition of various casting solutions.

Membrane	Casting Solution (wt%)			
	PSf	PSf-co-SBPSf	PEG	NMP
M0	15	0	0	85
M1	15	0	5	85
M2	13	2	0	85
M3	12	3	0	85
M4	11	4	0	85
M5	10	5	0	85

**Table 2:** Physical properties of resultant membranes.

Membrane	Porosity (%)	Mean Pore Size (nm)	Over Membrane Thickness (μm)	Skin Layer Thickness (μm)
M0	46.3 ± 0.6	3.9	110 ± 3	1.46 ± 0.4
M1	62.2 ± 0.3	13.4	106 ± 11	1.23 ± 0.4
M2	76.7 ± 0.4	5.6	109 ± 8	1.06 ± 0.7
M3	79.6 ± 0.2	6.1	105 ± 2	0.98 ± 0.2
M4	81.1 ± 0.3	5.8	106 ± 6	0.91 ± 0.6
M5	85.8 ± 0.5	6.5	103 ± 4	0.83 ± 0.4

## Data Availability

Not applicable.

## Supplementary Materials

The following are available online at https://www.mdpi.com/article/10.3390/membranes11120932/s1, Figure S1: SEM images of the membranes M0 (a), M1 (b), M2 (c), M3 (d), M4 (e) and M5 (f) surfaces after *E. coli.* adhesion test.
