# Supplementary material for: Zwitterionic Polysulfone Copolymer/Polysulfone Blended Ultrafiltration Membranes with Excellent Thermostability and Antifouling Properties
Source: Membranes (Basel). 2021 Nov 26;11(12):932. doi: 10.3390/membranes11120932 (PMC8707127; doi:10.3390/membranes11120932)
Supplement: Supplementary file 1 [file membranes-11-00932-s001.zip › membranes-1480705-supplementary.pdf]

Support information

Zwitterionic Polysulfone Copolymer/Polysulfone Blended Ultrafiltration Membranes with Excellent Thermostability and Antifouling Properties

Dalong Li [1,2,3,*], Changlu Gao [1], Xinyue Wang [1], Gang Wu [2,3], Jinghua Yin [2,3], Yudong Huang [4] and Xiuhua Sun [1,*]

1 School of Marine Science and Technology, Harbin Institute of Technology at Weihai, Weihai 264209, China; changlugao@163.com (C.G.); hitwhwxy@163.com (X.W.)
2 Shandong Weigao Group Co. Ltd., Weihai 264210, China; wugwego@163.com (G.W.); yinjhwego@163.com (J.Y.)
3 National Engineering Laboratory of Medical Implantable Devices & Key Laboratory for Medical Implantable Devices of Shandong Province, WEGO Holding Company Limited, Weihai 264210, China
4 School of Chemical Engineering and Technology, Harbin Institute of Technology, Harbin 150001, China; huangydhit@163.com
* Correspondence: lidalong@hit.edu.cn (D.L.); sunxh@hitwh.edu.cn (X.S.)

Experiment:

Anti-bioadhesion properties. *Escherichia coli* (*E. coli*) bacteria were used to evaluate the antibacterial characteristics of ultrafiltration membrane. In the experiment, all the membrane samples were sterilized by ultraviolet radiation for 30 min. To inoculate the bacteria, the sterilized membrane samples were immersed into 100 mL of the *E. coli* bacteria suspension at the concentration of 10^7 colony forming units per ml (cfu/mL) at 37 °C for 24 h. After the inoculation, the membrane samples were repeatedly washed with PBS. Then the inoculated bacterial on the membrane surfaces were fixed in a 2.5% (v/v) glutaraldehyde solution in PBS at 4 °C for 4 h. Finally, the membrane samples were washed with PBS and ethanol to remove remaining glutaraldehyde. After the samples were dried, the membrane surfaces were coated with Au for SEM observation (Zeiss MERLIN Compact, Germany).

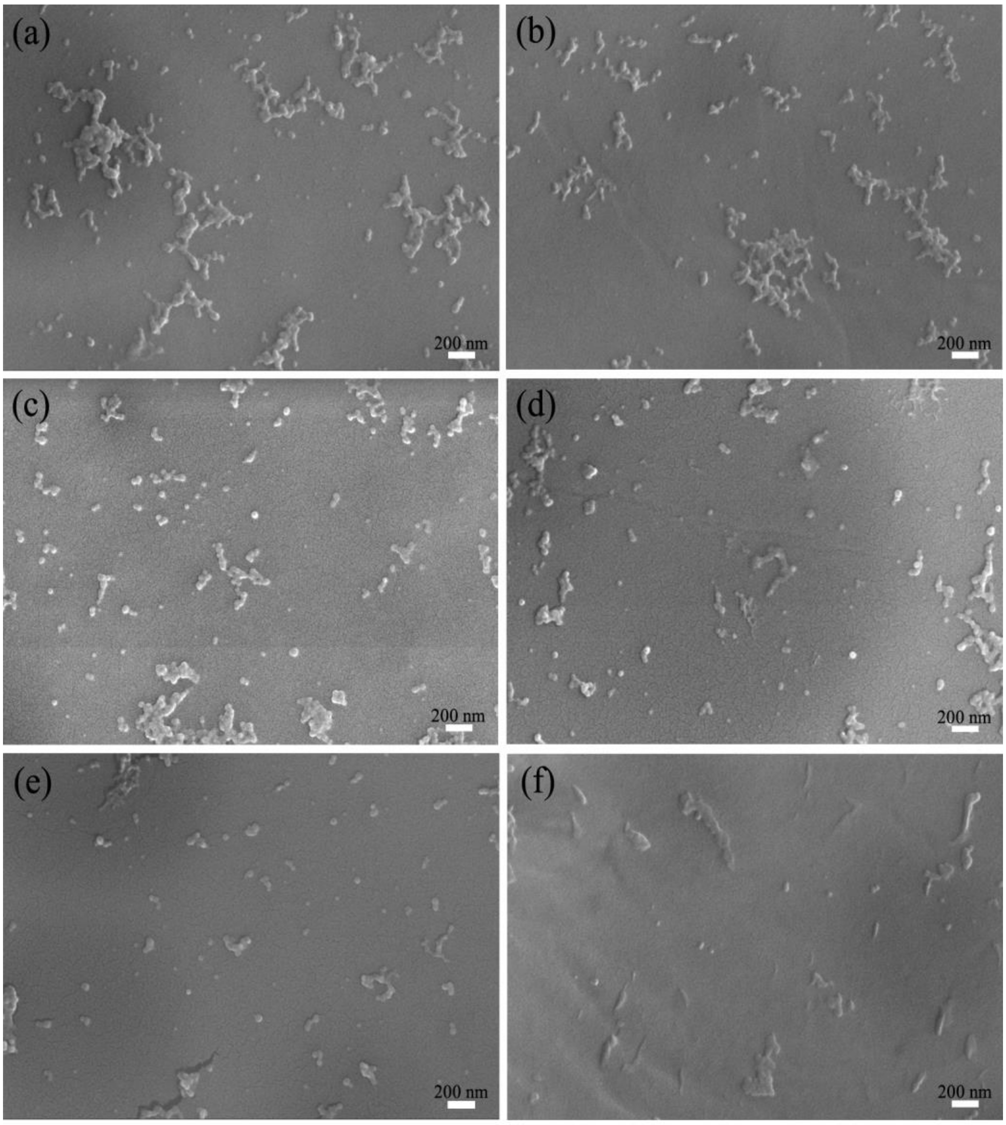

Figure S1. SEM images of the membranes M0 (**a**), M1 (**b**), M2 (**c**), M3 (**d**), M4 (**e**) and M5 (**f**) surfaces after *E. coli.* adhesion test
